# Practice of “Specialties” in Medicine

**Published:** 1867-01

**Authors:** 


### Editorial Department.

### Practice of "Specialties" in Medicine.

Though much has been written and said in our Society meetings and in the medical journals throughout the country, upon the subject of specialties, still the points in controversy remain as unsettled as ever—the real grounds of objection or favor are yet quite frequently misunderstood. At the last annual meeting of the Erie County Medical Society, a proposition was introduced with the view of obtaining the sense of the Society upon the question of admitting *specialists* to membership, and some physicians appeared to hold, that such practice is objectionable to such a degree, as to constitute in itself controlling objection. The question was waived, and no action taken upon it.

That a physician may devote his attention exclusively to any of the various departments of medical practice, and yet hold honorable membership in the profession is settled beyond all controversy, and no objection can be urged against any one who thus faithfully devotes his attention to the cure of special disease; it is not only unobjectionable, but praiseworthy and commendable.

The real objection which may with great reason be urged against the majority of "specialists," so calling themselves, consists in the unfounded claims they make to special qualification, experience and success, and the offensive, unprofessional, undignified and disgusting manner in which they parade their pretentions before the public. The country is filled with young, inexperienced, often uneducated and ignorant itinerant practitioners, some of whom have diplomas from respectable schools, but many, possessing only bogus titles, and all alike, practicing the customs of quacks. From this army of opposition and imposition, this heterogenious mass of incapacity and dishonesty, it is very difficult, if not impossible, to select any true, loyal, high-minded, worthy members of an honorable profession. The custom of newspaper advertising is often looked upon as one of the most objectionable features of this system of special imposition, but it is only a small item, necessary to make up the sum total of a complete imposter. Upon every occasion and in all places, the whole thought and being is enveloped by, and absorbed in, the controlling aim and purpose to deceive, and an honest desire to know the truth and practice accordingly, a firm purpose to render a just equivalent, and to speak truly, never intrudes itself into the aims and purposes of those who fill the columns of the daily papers with these offensive and absurd advertisements. It is not the advertising, which renders them so objectionable, but it is the little they know, and the amount they pretend ; it is what they say, and think, and do ; it is what they are

If a physician has been led by education, inclination or accident, to choose any department of medicine, and has made it his special study, directing his attention and observation to a complete knowledge of it, he may rightfully devote himself to his specialty, but it is prepostorous for a young man to commence his professional life, with the startling announcement to the public, that he "pays exclusive and special attention to ———— and is thus enabled to effect the most wonderful results," when in truth, the general practitioner of a few years' experience, knows much more of the diseases belonging to his "specialty" even, to say nothing of the general principles of medicine, and is vastly better qualified to manage diseases not only of that class, but of all other classes. The practice of specialties in this country, has thus far proved only a cunning dodge to obtain undeserved distinction ; and in all countries and everywhere, is beset by temptations which make it "easier for a camel to go through the eye of a needle" than for one of these, to maintain a just balance, rendering to Cæsar the things which are Cæsar's. If a physician practices only in diseases of the heart and lungs, it is remarkable how everybody who applies to him—all who come under his observation, come also into his department. Or, if the diseases of women receive special attention, what an astonishing number of females are found with uterine disturbances. If renal affections, or any other class of diseases are the objects of exclusive attention, the exclusive always means inclusive of everything exclusive. The true specialists are those only who have shown in patient study and by long practice an eminent fitness for special diseases or important trusts, and to these men alone can be safely and prudently entrusted the management of many of the more dangerous and difficult cases of disease, and their advice should be obtained upon the ground of special qualification ; they are the true specialists, and there are no others.

It is often said that the profession are opposed to specialists, but this is not true ; physicians everywhere respect and fellowship those who have advanced the science or practice of medicine or surgery in any of its departments. Let any man show an earnest, consistent devotion to medical knowledge, and he is respected by physicians the world over, and if he excels in any respect, his excellence is acknowledged. We have learned to be slow, in our acknowledgments of merit, that we may be correct; but we have always been ready, too ready, to listen to new modes of reasoning and new plans of practice, to every proposition of improvement. A great many have *suffered* from the *indifference* and *opposition* of the profession, suffered from adherance to the truth, from disbelief in prevalent opinions, from desire to practice according to the "dictates of their own consciences," from misrepresentation, jealousy and envy, have indeed become perfect through suffering, and now announce the wonderful results of the simplest operation, or the marvellous efficiency of some newly discovered compound; and humbly seek admission to respectable medical society, with the privilege of advertising (?) "Have large families and could not expect to obtain a livelihood with so many established physicians without *advertising*—no other way to get their names before the public." It is not much moment what is said or done with this matter of what we shall practice, admitted or not admitted to fellowship, holding professional credentials or not holding them, it is about the same thing in the end. The greatest concern with us, should be, that we are not quacks ourselves, and there is no danger of high-minded, intelligent physicians practicing otherwise than upon the strictest principles of professional courtesy. The others are of no importance one way or the other, they serve only as contrasts by which the real men of the profession may be better known, more admired andrespected. I have sometimes wondered that the notice of respectable physicians could be gained by the unimportant pretenders who prate and rattle upon the surface with such pretentious roar, but never approach or disturb the still waters of professional worth. They neither add or subtract from human knowledge, and may safely be left to live their brief period, to be succeeded by other anomalies; for though they mny increase and multiply, they never propagate their own species, there is an incessant and unending change.

